# Evolution in fluctuating environments: A generic modular approach

**DOI:** 10.1111/evo.14616

**Published:** 2022-10-12

**Authors:** Bnaya Steinmetz, Immanuel Meyer, Nadav M. Shnerb

**Affiliations:** ^1^ Department of Physics Bar‐Ilan University Ramat‐Gan IL 52900 Israel

**Keywords:** Competition, chance of ultimate fixation, fitness, fluctuations, varying environment

## Abstract

Evolutionary processes take place in fluctuating environments, where carrying capacities and selective forces vary over time. The fate of a mutant type and the persistence time of polymorphic states were studied in some specific cases of varying environments, but a generic methodology is still lacking. Here, we present such a general analytic framework. We first identify a set of elementary building blocks, a few basic demographic processes like logistic or exponential growth, competition at equilibrium, sudden decline, and so on. For each of these elementary blocks, we evaluate the mean and the variance of the changes in the frequency of the mutant population. Finally, we show how to find the relevant terms of the diffusion equation for each arbitrary combination of these blocks. Armed with this technique one may calculate easily the quantities that govern the evolutionary dynamics, like the chance of ultimate fixation, the time to absorption, and the time to fixation.

Competition between different types, at whatever scale of organization, is a key driver of evolutionary dynamics. The interplay between selective forces (if exist) and genetic drift governs the chance of adapted phenotypes to reach fixation, the rate in which deleterious alleles disappear through purifying selection and the fate of a neutral (e.g., synonymous) mutation. The simplest and most important scene in which these factors manifest themselves is the competition between a mutant type and a wild type. This case received a lot of theoretical and empirical attention, and many classical works are devoted to the analysis of its characteristics. In particular, attention was given to three important quantities: the chance of ultimate fixation Π, the mean time to absorption (either fixation or loss) $T_{A}$, and the mean time to fixation $T_{F}$ (Crow et al., 1970; Ewens, 2012; Kimura, 1962).

Most of the existing literature is focused on the case of a fixed environment, where demographic parameters and selective forces are time‐independent. However, all evidence shows that ecological and evolutionary processes are usually subject to wild environmental fluctuations which cause large changes in demographic rates and in the frequency of different types (Adler et al., 2006; Bergland et al., 2014; Fung et al., 2020; Kalyuzhny et al., 2014; Lande et al., 2003; Mellard et al., 2019; Usinowicz et al., 2012; White & Hastings, 2020). Some of these variations affect the selective forces, others change the overall size of a population; of course, these effects may be intertwined. Temporal variations may be periodic (seasonal) or stochastic, and they often yield highly nontrivial outcomes. In particular, in some cases temporal variations act to promote coexistence and protect polymorphism through mechanisms like the storage effect (Bertram & Masel, 2019; Chesson, 2000; Chesson & Warner, 1981; Chesson, 1982b; Usinowicz et al., 2017; Yi & Dean, 2013). By and large, competition in fluctuating environment admits a much richer phenomenology, and at the same time is much more difficult to analyze.

Even in experimental setups, the effective demographic rates vary over time. For example, in the famous Long‐Term Evolutionary Experiment of Lenski and coworkers (Good et al., 2017; Lenski et al., 1991) *E‐coli* strains compete in an apparently fixed environment: all flasks contain the same medium, nutrient concentration and temperature are kept fixed, and so on. Yet, every day (i.e., every 6‐7 generations) each population is diluted to about 1% of its size, then it undergoes an exponential growth phase and then saturates. Unlike a constant‐size population, in such experiments the chance of a rare mutation to establish depends very much on its chance to survive a few dilution steps while rare.

The importance of temporal variations was acknowledged and analyzed in many (mostly recent, but see Takahata et al. 1975; Takahata & Kimura 1979) theoretical works. Whal and Gerrish (Wahl & Gerrish, 2001), for instance, provided a thorough analysis of the two‐type competition dynamic in Lenski's experiment. Other authors dealt with other specific realizations: fixed population size with varying selection (Ashcroft et al., 2014; Cvijović et al., 2015; Danino & Shnerb, 2018; Hidalgo et al., 2017; Huerta‐Sanchez et al., 2008; Mustonen & Lässig, 2008; Meyer & Shnerb, 2018; Marrec & Bitbol, 2020), fixed selection with varying population size (Wienand et al., 2017, 2018), and so on. Despite all these efforts, a generically implementable methodology is still lacking.

Here, we would like to provide such a generic technique. Our method utilizes the diffusion approximation (DA), which is the standard tool in the analysis of stochastic systems (Karlin & Taylor, 1981). We consider a set of typical demographic processes: growth (logistic or exponential) or decline (logistic, exponential, or sharp dilution) of the total population and various competition scenarios (local or global) when the total population size is fixed. Each of these processes is a “building block,” and we calculate its effect on the frequency of the mutant type and its second moment. We then show how to construct the diffusion equation for an arbitrary sequence of these building blocks.

The general approach suggested in this paper is illustrated in Figures 1 and 2. In what follows we refer to the different panels of these figure, which illustrate the main steps required.

1. Panel (a) of Figure 1 is a cartoon of a possible process, which is characterized by two parameters: the total population size N(t) and the selection parameter s(t). Both parameters may vary through time. For example, as resource become more prevalent the total carrying capacity increases, and at the same time the relative fitness of the wild type and the mutant may (or may not) change. For the moment, our information is fully described by two given (but otherwise arbitrary) functions of time N(t) and s(t).
2. Panel (b) of Figure 1 describes a “model” of the actual process. Now N(t) and s(t) are not arbitrary functions of time, instead the process is modeled as a collection of growth, decline and fixed population periods. As explained below, we allow a lot of flexibility for these elementary building blocs. For example, growth may be exponential or logistic, where its rate, its duration and the selective forces during growth are still arbitrary. An abstraction of this type is essential, of course, to any mathematical analysis of a real‐life process, and we believe that our collection of elementary building blocks may account for the actual dynamic in most of the relevant scenarios.
3. Once a model is established, a “representative segment” of duration T has to be identified (panel c of Figure 1 and Figure 2). Such a segment must provide a fair representation of the whole process, so its building blocks composition and strength (durations, selection and so on) should reflect the main features of the process. If the environment is periodic, the representative segment is a single period. In aperiodic setups the quality of the approximation may be evaluated by examining the robustness of the result to the addition of more blocks.
4. Let us focus, now, on the *i*‐th building block and its parameters (duration $\tau_{i}$, selection parameter $s_{i}\left(t\right)$, $N_{i}\left(t\right)$, and so on, as illustrated in Figure 2). To implement the diffusion equation one would like to calculate two quantities that characterize its effect on the frequency of the mutant type *x*. First, the “velocity,” which is the mean change in the frequency $\mu_{i}=\bar{\Delta x_{i}/\tau_{i}}$. Second, the “diffusion” parameter, which is simply the second moment, $\sigma_{i}^{2}=\bar{\Delta^{2}x_{i}/\tau_{i}}$. In Table 2 below, we provide $\mu_{i}\left(x\right)$ and $\sigma_{i}^{2}\left(x\right)$ for each building block in our collection.
5. Given $\mu_{i}\left(x\right)$ and $\sigma_{i}^{2}\left(x\right)$ for each block, one should find μ(x) and $\sigma^{2}\left(x\right)$ for the entire representative segment. This step in explained in section 4.
6. Finally, μ(x) and $\sigma^{2}\left(x\right)$ of a representative segment are given, these quantities are plugged in the diffusion equations for the chance of ultimate fixation, time to absorption and time to fixation (Equations (1), (2), (3) below). The emerging second order (homogenous or inhomogeneous) differential equations are easily solvable, either numerically or analytically, and we provide these solutions in an integral form.

**Figure 1:**
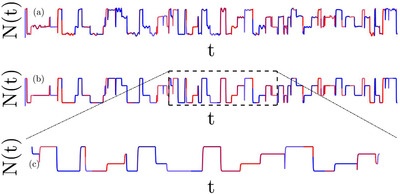
Selecting a representative segment. A cartoon of a possible environmental process is shown in panel (a): the total population *N* fluctuates with time, and periods in which the mutant is beneficial (red) are followed by periods of advantageous wild type (blue). The process itself may be very complicated, and as a first approximation one has to replace it by another process made of our building blocks: growth periods (exponential or logistic), decline periods (exponential, logistic or sharp) and fixed population competition periods (global or local competition, periodic or stochastic variations of selective forces), as shown in panel (b). To implement the procedure suggested here a representative segment [panel (b), dashed rectangle, magnified in panel (c)] is chosen and analyzed. The order of these building blocks is arbitrary, any block may be followed by any other block, see Section 6 below. A simple example of a representative segment is provided in Figure 2; a more intricate case is presented in panel (a) of Figure 4.

**Figure 2:**
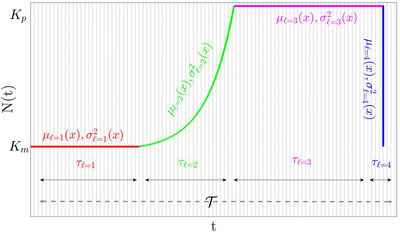
A possible representative segment, consists of four ”building blocks”. Here, a constant population competition with $K_{m}$ individuals (red) is followed by exponential growth to $K_{p}$ (green), a constant population competition phase at $K_{p}$ (magenta) and a sharp decline (blue). Two types of dynamic that correspond to this cartoon are analyzed in Section 6 (lines 1 and 2 of Table 3). The basic timescale of the problem (say, one day or one month), τ_0_, is indicated by thin vertical dashed lines. Blocks are numbered in order of occurrence, using the index ℓ, and the duration of each block (in τ_0_ units) is $\tau_{\ell }$. Finally, the duration of the whole representative segment (again, in units of τ_0_) is T. In Section 6 below, we read the functions $\mu_{\ell }\left(x\right)$ and $\sigma_{\ell }^{2}\left(x\right)$ from the relevant lines of Table 2, and calculate the combined parameters of the whole segment, μ(x) and $\sigma^{2}\left(x\right)$, using the procedure presented in Section 4 below.

**Table 1:** Glossary

Term	Description
*N*	total number of individuals in the community at a given time.
*n*	number of mutant individuals.
*x*	frequency of the mutant type, x=n/N (1−x is the wild type frequency).
τ_0_	an arbitrary (conveniently chosen) time. Times and rates are defined in units of τ_0_.
*s*_0_	time‐independent component of the fitness (competition at constant population).
γ	amplitude of fitness fluctuations (competition at constant population).
δ	mean number of birth‐death events before selection changes (competition at constant population).
$g\equiv \delta \gamma^{2}/\left(2N\right)$	the strength of selection fluctuations (competition at constant population).
τ	duration (in units of τ_0_) of a constant population state.
*r*_0_	rate of events in the constant population state, the mean number of events per τ_0_.
$\lambda ,r_{1}..r_{4}$	growth and decline rates, measured in units of $1/\tau_{0}$
$s_{g}$	selection parameter for logistic population growth.
$s_{d}$	selection parameter for logistic decline and abrupt population decline
$t_{g,d}$	growth (decline) time in the logistic growth (decline) scenario, in units of τ_0_
η=1+N/δ	useful parameter
T	The duration of a representative segment (in units of τ_0_)
$K_{p},K_{m}$	high and low target carrying capacities, used to describe growth and decline events.

**Table 2:** The contribution of each building block

*i*	state	$\mu_{i}\left(x\right)$ (during τ_0_)	$\sigma_{i}^{2}\left(x\right)$ (during τ_0_)	duration $\tau_{i}$ (in τ_0_ units)
1	population equilibrium local competition, periodic selection changes	$\frac{r_{0}}{N}x\left(1-x\right)s_{0}$	$\frac{r_{0}}{N^{2}}2x\left(1-x\right)$.	τ
2	population equilibrium local competition, stochastic selection changes	$\frac{r_{0}}{N}x\left(1-x\right)\left[s_{0}+g\left(1-2x\right)\right]$	$\frac{2r_{0}x\left(1-x\right)\left[1+gNx\left(1-x\right)\right]}{N^{2}}$.	τ
3	population equilibrium global competition, periodic selection changes	$\frac{r_{0}}{N}x\left(1-x\right)\left[s_{0}+\frac{\gamma^{2}}{2}\left(1-2x\right)\right]$	$\frac{r_{0}}{N^{2}}2x\left(1-x\right)$.	τ
4	population equilibrium global competition, stochastic selection changes	$\frac{r_{0}}{N}x\left(1-x\right)\left[s_{0}+\eta g\left(1-2x\right)\right]$	$\frac{2r_{0}x\left(1-x\right)\left[1+gNx\left(1-x\right)\right]}{N^{2}}$.	τ
5	population growth from $K_{m}$ to $K_{p}$ exponential	$\frac{\left(r_{1}-r_{2}\right)x\left(1-x\right)}{1+r_{1}x+r_{2}\left(1-x\right)}$	$x\left(1-x\right)\left[\frac{1}{K_{m}}-\frac{1}{K_{p}}\right]\frac{xr_{2}+\left(1-x\right)r_{1}}{\log(K_{p}/K_{m})}$	$\frac{\log(K_{p}/K_{m})}{xr_{1}+\left(1-x\right)r_{2}}$
6	population growth from $K_{m}$ to $K_{p}$ logistic	$\frac{x(1-x)}{t_{g}}\frac{{\left(K_{p}/K_{m}\right)}^{s_{g}}-1}{1-x+x{\left(K_{p}/K_{m}\right)}^{s_{g}}}$	$\frac{x(1-x)}{t_{g}}\left(x\left(1-x\right){\left[\frac{{\left(K_{p}/K_{m}\right)}^{s_{g}}-1}{1-x+x{\left(K_{p}/K_{m}\right)}^{s_{g}}}\right]}^{2}+\frac{1}{K_{m}}-\frac{1}{K_{p}}\right)$	$t_{g}$ (Eq. 10)
7	population decline from $K_{p}$ to $K_{m}$ exponential	$\frac{\left(r_{4}-r_{3}\right)x\left(1-x\right)}{1-r_{3}x-r_{4}\left(1-x\right)}$	$x\left(1-x\right)\left[\frac{1}{K_{m}}-\frac{1}{K_{p}}\right]\frac{xr_{4}+\left(1-x\right)r_{3}}{\log(K_{p}/K_{m})}$	$\frac{\log(K_{p}/K_{m})}{xr_{3}+\left(1-x\right)r_{4}}$
8	population decline from $K_{p}$ to $K_{m}$ logistic	$\frac{x(1-x)}{t_{d}}\frac{{\left(K_{p}/K_{m}\right)}^{s_{d}}-1}{1-x+x{\left(K_{p}/K_{m}\right)}^{s_{d}}}$	$\frac{x(1-x)}{t_{d}}(x\left(1-x\right){\left[\frac{{\left(K_{p}/K_{m}\right)}^{s_{d}}-1}{1-x+x{\left(K_{p}/K_{m}\right)}^{s_{d}}}\right]}^{2}+|\frac{1}{K_{m}}-\frac{1}{K_{p}}\left|\right)$	$t_{d}$ (Eq. 12)
9	population decline from $K_{p}$ to $K_{m}$ abrupt	$s_{d}x\left(1-x\right)\frac{K_{p}}{K_{m}}\frac{1}{t_{sh}}$	$x\left(1-x\right)\left[\frac{1}{K_{m}}-\frac{1}{K_{p}}\right]\frac{1}{t_{sh}}$	$t_{sh}\to 0$

The diffusion approximation is based on a second‐order expansion of the relevant quantities (for example, the chance of ultimate fixation Π(x), where *x* is the frequency of the mutant type), and the small parameter is Δx, the change in *x* during each step. Therefore, our technique requires that the changes in *x* in a single representative segment T are small enough, otherwise the expansion cannot be justified. In the “quenched” limit, that is, when the time to fixation is shorter than the persistence time of the environment, there is no point in averaging over many environmental states, as the fate of most mutants is determined by the environmental condition at the time of its birth (Cvijović et al., 2015; Mustonen and Lässig, 2008). Even when $T_{F}$ is indeed very large, the diffusion approximation may break down because the changes in *x* per representative segment are too large (Assaf & Meerson, 2017; Elgart & Kamenev, 2004; Kessler & Shnerb, 2007; Ovaskainen & Meerson, 2010; Pande & Shnerb, 2020). Still, in many cases the representative segment appears to be short enough. For example, in Lenski's dilution experiment a segment is one day (about 6.6 generations), but fixation requires about 1000 generations and the changes in frequency during a day are usually small (Good et al., 2017). Even beyond its range of applicability, in some cases the diffusion approximation (DA) produces quite accurate results (Parsons et al., 2010), and in all cases it provides, if not exact numbers, at least an intuitive understanding of the dynamic and its main features.

The expressions we provide for the building blocks are quite flexible. For example, when the logistic growth is considered, the formula covers any initial and final population state and any growth rate (or equivalently any duration of the growth). It also takes into account possible selective features (differences in growth rates). In the saturation phase (constant population) selective forces may vary either stochastically or periodically, competition may be either global or local and so on.

This paper is organized as follows. In the next section, we present the DA formulas for the important quantities considered through this paper. Section 3 is devoted to a description of the dynamic in each of the building block processes and the corresponding functions $\mu_{i}$ and $\sigma_{i}^{2}$ are presented in Table 2. In Section 4, we explain how to assemble these building blocks to obtain the coefficients of the diffusion equation. The solutions of these equations are provided in Section 5, along with a few worked examples in Section 6. Finally, we summarize the result and discuss their possible implications.

## The Diffusion Approximation in Varying Environments

As mentioned, the three important quantities considered through this paper are,

1. The chance of ultimate fixation Π(x), which is the probability that the mutant type (at frequency *x*) reaches fixation.
2. The mean time to absorption $T_{A}\left(x\right)$. For a given mutant population of frequency *x*, this is the mean time until it reaches either fixation or extinction.
3. The mean time to fixation $T_{F}\left(x\right)$, which is the expected duration before fixation, where the average is taken over all histories that end up in fixation (not over histories in which the mutation is lost).

The diffusion approximation is the standard tool in the analysis of stochastic processes, like those considered here (Ewens, 2012; Karlin & Taylor, 1981; Redner, 2001). Using DA, one translates a discrete difference equation (like the Master equation) into a differential equation by expanding the relevant quantities to second order in frequency or abundance differences. For example, in a population of *N* individuals one may ask what is $\Pi_{n}$, the chance of a mutant type with *n* individuals to reach fixation at n=N before going extinct. When DA is implemented the object considered is Π(x), where the frequency x=n/N is regarded as a continuum variable. This quantity is plugged into the appropriate discrete equation, and Π(x+Δx) is expanded to second order in Δx, the resulting differential equation and boundary conditions take the form,

(1) $$\mu \left(x\right)\Pi^{\prime }\left(x\right)+\frac{\sigma^{2}\left(x\right)}{2}\Pi^{\prime \prime }\left(x\right)=0\;\Pi \left(0\right)=0\;\;\mathrm{and}\;\;\Pi \left(1\right)=1.$$

where primes represent derivatives with respect to *x*. Here, μ(x) is the “velocity” (mean change in *x*, Δx, per unit time τ_0_) and $\sigma^{2}\left(x\right)$ is the “diffusion” term (variance of Δx per the same unit time). The choice of an appropriate unit time τ_0_ is discussed in the next section.

Similarly, the equation for the mean time to absorption $T_{A}$ (time is measured in τ_0_ units) is,

(2) $$\mu \left(x\right)T_{A}^{\prime }\left(x\right)+\frac{\sigma^{2}\left(x\right)}{2}T_{A}^{\prime \prime }\left(x\right)=-\tau_{0}\;T_{A}\left(0\right)=0\;\;\mathrm{and}\;\;T_{A}\left(1\right)=0.$$

To calculate the mean time to fixation $T_{F}\left(x\right)$ one must define $Q\left(x\right)=T_{F}\left(x\right)\Pi \left(x\right)$, where Q(x) satisfies,

(3) $$\mu \left(x\right)Q^{\prime }\left(x\right)+\frac{\sigma^{2}\left(x\right)}{2}Q^{\prime \prime }\left(x\right)=-\Pi \left(x\right)\tau_{0},\;Q\left(0\right)=0\;\;\mathrm{and}\;\;Q\left(1\right)=0.$$

Once Q(x) is known, time to fixation is obtained through $T_{F}\left(x\right)=Q\left(x\right)/\Pi \left(x\right)$.

The problems thus reduces to homogenous (Equation 1) or inhomogeneous (Equations 2 and 3) first order linear differential equations for the derivatives of the required quantities (e.g., $\Pi^{\prime }\left(x\right)$). These equations may be solved quite easily by two integrations, as explained in Appendix C, see Equations ((19), (20), (21), (22), (23)) below. If the expressions obtained are too complicated [that depends on the form of the functions μ(x) and $\sigma^{2}\left(x\right)$], simple numerical integration may be implemented. Therefore, the nontrivial task is to find the appropriate expressions for the moments μ(x) and $\sigma^{2}\left(x\right)$ (technically speaking, $\sigma^{2}\left(x\right)$ is the variance of Δx. However the diffusion approximation requires μ^2^ to be much smaller than the second moment, so the variance and the second moment are almost identical, therefore we refer to this quantity as the second moment).

In a fixed environment, the rates of events (and hence the transition probabilities) do not change in time. Stochasticity in a fixed environment is “demographic”—it reflects the endogenous stochastic characteristics of the birth–death process that are uncorrelated among different individuals. In that case one may calculate μ(x) and $\sigma^{2}\left(x\right)$ via the first two moments of the transition probabilities $W_{n\to n\pm 1}$ for an elementary step, for example, for a single birth–death event.

In a varying environment, demographic rates and transition probabilities are time‐dependent. This reflects large‐scale events, such as variations in temperature or in precipitation levels, that affect at the same time all (or almost all) individuals. Most importantly, environmental variations are typically correlated in time so there are many elementary birth‐death events before the environment changes substantially. In that case, one must calculate μ and σ^2^ from $W_{n(t)\to n(t+T)}^{k}$, where the index *k* reflects the state of the environment and T is an intermediate timescale which is longer than the correlation time of the environment. This procedure ensures that all different environmental states are sampled with their appropriate weights, and that correlations are taken into account. As discussed above, T must be also short enough such that Δx during this timescale is small, otherwise the second order expansion required for the diffusion approximation becomes insufficient. Usually, for large *N* one may find such a timescale. In fact, the existence of this intermediate timescale is what allows us to write equations for Π(x), say, and to neglect the dependence of the chance of ultimate fixation on the initial environmental state. Because the system visits many different environmental states before *x* changes substantially, we can average over all initial environmental conditions (Danino et al., 2018).

Note that the appropriate Fokker–Planck (forward Kolmogorov) equation, which describes the dynamic of P(x,t)dx, the chance to find the system at the vicinity of *x*, is,

(4) $$\frac{\partial }{\partial t}P\left(x,t\right)=\frac{\partial^{2}}{\partial x^{2}}\left(\frac{\sigma^{2}\left(x\right)}{2}P\left(x,t\right)\right)-\frac{\partial }{\partial x}\left(\mu (x)P(x,t)\right).$$

So this equation is also given once μ(x) and $\sigma^{2}\left(x\right)$ are known. Since we would like to take demographic stochasticity into account, P(x) never admits a normalizable steady‐state solution, so we do not elaborate on this equation. However, for some quantities of interest (Green's functions, quasi‐stationary solutions) implementation of the Fokker–Planck equation is required, in which case our results are applicable as well.

## The Building Blocks

In this section we describe our building blocks: a set of elementary processes that typically appear in competition dynamics. We consider situations where the population (total number of individuals) grows or declines, as well as equilibrium dynamics where the total population size is fixed and competition is a zero‐sum game. A glossary of the parameters used is provided in Table 1. The results are shown in Table 2 and the detailed calculations that lead to these results are presented in Appendix A. Some examples are given in Appendix A.

In Section 4, we will show how to incorporate these building blocks into the diffusion equation. To do that one needs not only the formulas for $\mu_{i}$ and $\sigma_{i}^{2}$ for each block type, but also their durations that determine the relative contribution of each block to the functions that govern the diffusion equation, μ(x) and $\sigma^{2}\left(x\right)$.

Our fundamental time unit is τ_0_, that may be a day, a year, or any other convenient time interval. All rates in the system must be defined with respect to τ_0_. In Table 2, we used a set of rate constants: $\left\{r_{0},r_{1}..r_{4},\lambda \right\}$. Below we explain how each of these constants is related to the rate of elementary birth‐death events. Of course the final diffusion equation is independent of the choice of the elementary timescale τ_0_, but once τ_0_ is defined, the rate constants must be consistent with this choice.

### CONSTANT POPULATION

When the total population is fixed with *N* individuals, we consider four different competition scenarios. We distinguish between two competition modes, local and global, and between two modes of environmental (and corresponding relative fitness) variations, stochastic and periodic. These four scenarios may yield different dynamical behaviors, and the derivation of the results presented in lines 1–4 of Table 2 is provided in Meyer et al. (2022).

The distinction between local and global competition is quite important. Environmental variations may facilitate coexistence (protect polymorphism) via the storage effect (Bertram & Masel, 2019; Chesson & Warner, 1981), but this effect does not manifest itself under local competition, as in that case there is no covariance between competition and environment (Meyer et al., 2022). In intermediate cases, when competition is restricted to a single deme or a single patch, the storage effect may provide a mechanistic explanation for balancing selection, so these cases have to be modeled with global competition.

#### Local competition

Local competition scenarios are typical in animals populations, where individuals wander around searching for food or territory, and an encounter between two individuals may end up struggling for these goods. To model that, we implement the pairwise comparison scheme suggested in Volkov et al. (2003); Traulsen et al. (2007). Two individuals are chosen at random in each time step for a duel, the loser dies and the winner reproduces so the total number of individuals is kept fixed. For two species competition, when the frequency of the mutant type is x≡n/N, the chance of an interspecific duel is 2x(1−x). We use *s* as the selection parameter, so an individual's fitness is exp(s). If $e^{s_{1}\left(t\right)}$ is the fitness of the mutant type and $e^{s_{2}\left(t\right)}$ is the fitness of the wild type, the chance of the mutant to win an interspecific duel is,

(5) $$f_{+}=\frac{e^{s_{1}\left(t\right)}}{e^{s_{1}\left(t\right)}+e^{s_{2}\left(t\right)}},$$

and the chance of the wild type is $1-f_{+}$. Without loss of generality we assume the dynamic of the relative selection to satisfy $s\left(t\right)\equiv s_{1}\left(t\right)-s_{2}\left(t\right)=s_{0}+\zeta \left(t\right)$, where *s*_0_ is the mean relative selection and ζ(t) is a zero‐mean stochastic process.

Given the (arbitrarily chosen) elementary time interval τ_0_, we define the overall rate of events as *r*_0_, so the mean number of duels in each τ_0_ interval is *r*_0_. After every elementary birth‐death event, with probability 1/δ we pick ζ at random, so the dwell times of the environment are distributed geometrically and its mean corresponds to δ elementary events. Accordingly, in τ_0_ units the dwell time is $\delta /r_{0}$. When the environment changes, ζ is picked from a uniform distribution with zero mean and variance γ^2^. In periodic systems ζ flips its sign after δ steps.

To provide a numerical example, let's consider a population of N=100 individuals for which a duel happens every two days, environment switches every 14 days and the length of the whole block is 50 weeks. If τ_0_ is chosen to be a single day, then $r_{0}=1/2$, δ=7 and the number of τ_0_ intervals in this segment is τ=50×7=350.

The effect of these dynamics on the “velocity” per τ_0_,

(6) $$\mu \equiv \frac{\bar{\Delta x}}{\tau_{0}},$$

and on the associated second moment

(7) $$\sigma^{2}\equiv \frac{\bar{{\left(\Delta x\right)}^{2}}}{\tau_{0}},$$

are given in rows 1 (for periodic dynamic) and 2 (stochastic dynamic) of Table 2. For a duration τ the total change in *x* is $\mu_{i}\tau$ and the expected value of ${\left(\Delta x\right)}^{2}$ is $\sigma_{i}^{2}\tau$.

#### Global competition

In many populations individuals produce seeds (or larvae etc.) that disperse over long distances, or otherwise the relevant resources undergo fast diffusion. When an individual dies the competition for the released resource (room occupied by an adult tree, say, or the free nutrients in bacterial colonies) is more or less distance independent or global. Global competition is the scenario considered in the standard models of Wright‐Fisher and Moran (Ewens, 2012).

Here we implement a Moran process  (Moran, 1958): one individual is chosen at random to die, and its slot is recruited by an offspring of another individual with a chance proportional to its fitness. Accordingly, the chance of the mutant type (of frequency *x*) to increase its abundance by one is the chance that a wild type was chosen to die, 1−x, multiplied by the probability of the mutant to capture the empty slot,

(8) $$f_{+}=\frac{xe^{s_{1}\left(t\right)}}{xe^{s_{1}\left(t\right)}+\left(1-x\right)e^{s_{2}\left(t\right)}}.$$

Similarly, the chance of the mutant type to lose one individual is $x(1-f_{+})$.

The dynamics of s(t) (periodic or stochastic) are identical to those considered in case of local competition, and the numerical example provided above (for *r*_0_, τ_0_ etc.) is applicable in this case as well. The relevant $\mu_{i}$ and $\sigma_{i}^{2}$ are presented in rows 3 (periodic) and 4 (stochastic) of Table 2.

### VARYING POPULATION

During the evolutionary process the total population may grow or decline. This reflects improvement, or deterioration, of the environmental conditions that increase or decrease the overall carrying capacity. Such events may have different influence on the mutant and on the wild type: one type may admit features that allow it to utilize the new opportunities better than its opponent, or may be less vulnerable to periods of increased stress. Selective forces during growth and decline may differ from the selective forces during periods of constant population. Therefore, we define other independent parameters ($r_{1},r_{2},r_{3},r_{4},s_{g}$ and $s_{d}$), to denote the selective differences during growth and decline.

When resources become more abundant, or when an external event (like an epidemic) puts extra stress on populations, the steady‐state competition described above plays only a minor role in the dynamic. Other features, like maximum fecundity or resistance to pathogens, are more important. Therefore, we neglect steady‐state competition processes during the periods in which the total population changes.

Growth and decline may be characterized by three sets of parameters: population initial and final sizes, the rates of growth and decline and the length of growth and decline periods. Once two of these parameters are set, they determine the third one. Here we parameterize the process using the first two sets of parameters, namely growth or decline rates and the initial and final population size. From these, we derive the corresponding timescale associated with the (growth or decline) block. When the processes are exponential, the calculation of the relevant timescale $\tau_{i}$ is trivial. When a population grows or declines logistically it approaches exponentially its asymptotic value (the new carrying capacity), so the time required is formally infinite. Here we used the standard engineers convention and define the end of the growth process when the population size is within ε from the target capacity, with ε=exp(−5)≈0.67% of the target capacity.

#### Population growth

When the environmental conditions suddenly or gradually improve (more food, increase in habitat size) the carrying capacity increases. The overall growth rate (total birth minus total death) becomes positive until the population reaches the new equilibrium. We denote the low carrying capacity as $K_{m}$ and the high value as $K_{p}$.

Two characteristic cases were analyzed. When the growth is logistic, the growth rate declines linearly as the population approaches $K_{p}$ and vanishes at $K_{p}$. In many other systems the growth rate is almost independent of the population size and drops sharply to zero once the total population reaches $K_{p}$. In the logistic growth case, the fitness difference is defined as $s_{g}$. Logistic growth is a prototype of processes in which the carrying capacity grows gradually and the two types compete for the new recruitment events. Exponential dynamic characterizes the cases in which the two types grow independently until the total population reaches a new upper bound.

Our model for **“**exponential” growth with growth rate *r*_1_ (for mutant) and *r*_2_ (for wild type) is as follows. In every time interval τ_0_ the number of offspring produced by *n* mutant individuals is picked from a binomial distribution with *n* trials where the chance of success is *r*_1_, $B(n,r_{1})$, so $n\to n+B(n,r_{1})$. The same procedure is applied to the N−n wild type individuals, so $N-n\to N-n+B(N-n,r_{2})$. Time intervals τ_0_ must be taken small enough, such that *r*_1_ and *r*_2_ (the number of offspring per individual per τ_0_) are much smaller than one. The resulting formulas for $\mu_{i}\left(x\right)$ and $\sigma_{i}{\left(x\right)}^{2}$ are provided in line 5 of Table 2.

When the growth is “logistic,” N(t) satisfies

(9) $$\frac{dN(t)}{dt}=\lambda N\left(1-\frac{N}{K_{p}}\right)\;N\left(0\right)=K_{m}.$$

In our simulations, some small enough Δt is chosen (we implemented Δt=0.1/N), N(t) is incremented according to Eq. (9) using a simple Euler integration technique and the number of new recruits, ΔN=N(t+Δt)−N(t), is rounded to the nearest integer greater than or equal to it. ΔN is then divided between the mutant and the wild type based on their abundance and fitness: the mutant population increases according to $n\to n+B(\Delta N,x+s_{g}x\left(1-x\right))$ and all other new recruits are wild‐type individuals. The relevant formulas are presented in line 6 of Table 2.

As explained, the duration of the process is defined as the time in which the logistically growing population that satisfies Eq. (9) reaches Kp[1−ε]. A simple solution yields,

(10) $$t_{g}=\frac{\ln(c_{1}\left[Kp/Km-1\right]}{\lambda },$$

where $c_{1}=\left(1-\varepsilon \right)/\varepsilon \approx 150$. λ is the number of new slots opened per individual per τ_0_, and thus $t_{g}$ is given, again, in units of τ_0_.

#### Population decline

The decline of a population may be the result of a sudden catastrophe (earthquake, the impact of a meteor, hunting, epidemic, dilution in transfer experiments), or of gradual resource depletion. Here, we consider three characteristic cases: a sudden drop (as in transfer experiments), exponential decrease, and logistic decline.

**Exponential** decrease is parameterized by *r*_3_ (the decay rate of the mutant, which is the chance of an individual to die during τ_0_) and *r*_4_ (the decay rate of the wild type population). In every time step $n\to n-B(n,r_{3})$ and $N-n\to N-n-B(N-n,r_{4})$. The outcomes are presented in line 7 of Table 2.

In case of **logistic** decline, the total population satisfies,

(11) $$\frac{dN(t)}{dt}=\lambda N\left(1-\frac{N}{K_{m}}\right)\;N\left(0\right)=K_{p}.$$

The total death toll per Δt is ΔN=N(t)−N(t+Δt), (rounded to the nearest integer lower than it). The mutant population declines by a random number picked from a binomial distribution with ΔN trails and a chance of success $x-s_{d}x\left(1-x\right)$, all other death event occur in the wild type population. Results are presented in line 8 of Table 2.

Again, the duration of the process is the time required for the population in Eq. (11) to reach $K_{m}\left[1+\varepsilon \right]$. Therefore,

(12) $$t_{d}=\frac{\ln(c_{1}\left[1-Km/Kp\right]}{\lambda },$$

Finally, in case of **sharp decline** (such as an abrupt dilution) from $K_{p}$ to $K_{m}$, the number of mutant survivors is taken from $B(n,s_{d}+K_{m}/K_{p})$ and the number of wild type survives is $B(N-n,K_{m}/K_{p})$. Here we assumed $s_{d}K_{p}\ll 1$, otherwise the number of mutants has a strong effect on the result of the dilution. Results for that case are presented in line 9 of Table 2. The time required for the decline is by definition negligible; for consistency we denote it by $t_{sh}$, but the contribution of the dilution to Δx and to its moments is independent of $t_{sh}$.

## From the Building Blocks to the Diffusion Equation

Once a representative segment is identified, it typically contains a few different blocks (growth, constant population, and so on, see Figure 1). Some of these building blocks may appear more than once. We would like to distinguish between the specific indices associated with different block types (i=6, for example, is the logistic growth, as in Table 2) and indices associated with a given occurrence of a block in the representative segment, ℓ. For example, if we have in a representative segment five blocks—constant population (local, periodic), exponential growth, constant population (local, periodic), logistic decline and finally global‐stochastic constant population block—then the *i* indices are 1,5,1,8,4, but the ℓ index refers to blocks in the order in which they appear in the segment, ℓ=1,2,3,4,5. The maximum value that ℓ takes in a given segment, *m*, is simply the number of blocks in the representative segment.

Once $\mu_{\ell }\left(x\right)$ and $\sigma_{\ell }^{2}\left(x\right)$ of each building block ℓ are given, we can ensemble them to calculate the functions that govern the diffusion equation, namely $\mu_{m}\left(x\right)$ and $\sigma_{m}^{2}\left(x\right)$. The derivations for the following expressions are presented in Appendix B.

First, let us define the total duration of the representative segment. For a segment with *m* blocks,

(13) $$T_{m}=\sum_{\ell =1..m}\tau_{\ell }.$$

The function, $\sigma_{m}^{2}\left(x\right)$, is simply an additive weighted sum of all the $\sigma_{\ell }^{2}$s,

(14) $$\sigma_{m}^{2}\left(x\right)=\frac{1}{T_{m}}\sum_{\ell =1..m}\sigma_{\ell }^{2}\left(x\right)\tau_{\ell }.$$

The mean displacement $\mu_{m}\left(x\right)$ is obtained from the $\mu_{\ell }$ functions of the individual blocks through a recursive formula (here we assume that different blocks are uncorrelated, otherwise one cannot take the average over Δx for each block separately). For a single block *i*, $\mu_{m}\left(x\right)=\mu_{i}\left(x\right)$. For two blocks (m=2, e.g., logistic growth i=6 followed by sharp decline i=9),

(15) $$\mu_{1..2}\left(x\right)=\frac{\mu_{\ell =1}\left(x\right)\tau_{\ell =1}+\mu_{\ell =2}\left(x\right)\tau_{\ell =2}+\tau_{\ell =1}\mu_{\ell =1}\left(x\right)\tau_{\ell =2}\frac{d\mu_{\ell =2}\left(x\right)}{dx}}{\tau_{\ell =1}+\tau_{\ell =2}}.$$

In this specific example $\mu_{\ell =1}=\mu_{i=6}$ and $\mu_{\ell =2}=\mu_{i=9}$.

In general, adding a block ℓ after ℓ−1 other blocks yields,

(16) $$\mu_{1..\ell }\left(x\right)=\frac{\mu_{1..\ell -1}\left(x\right)T_{\ell -1}+\mu_{\ell }\left(x\right)\tau_{\ell }+T_{\ell -1}\mu_{1..\ell -1}\left(x\right)\tau_{\ell }\frac{d\mu_{\ell }\left(x\right)}{dx}}{T_{\ell }}.$$

After taking into account all *m* blocks inside the representative segment, $\mu_{m}\left(x\right)$ and $\sigma_{m}^{2}\left(x\right)$ are the μ(x) and the $\sigma^{2}\left(x\right)$ one has to put into Eqs. (1‐3).

The μ terms, as detailed in Table 2, all vanish when the relevant relative selection parameter ($s_{0},s_{g},s_{d},r_{1}-r_{2}$ etc.) vanishes. In many cases one consider a scenario with weak selective forces, neglecting high order terms in these parameters. When third order (proportional to $s_{0}^{2}$ or $s_{0}s_{d}$ etc.) and higher order terms are negligible, Eq. (16) simplifies to the time averaged value for μ(x),

(17) $$\mu_{m}\left(x\right)=\frac{1}{T_{m}}\sum_{\ell =1..m}\mu_{\ell }\left(x\right)\tau_{\ell }.$$

## Solutions of the Diffusion Equations

Once Eqs. (1‐3) are given, with an explicit form for the functions μ(x) and σ(x), the problem may be solved, either analytically or numerically, by introducing an appropriate integrating factor (Karlin & Taylor, 1981). This approach is explained in Appendix C.

We first define I(x),

(18) $$I\left(x\right)=\exp\left[-\int_{0}^{x}\frac{2\mu (t)}{\sigma^{2}\left(t\right)}dt\right].$$

The solution for the chance of ultimate fixation then takes the form,

(19) $$\Pi \left(x\right)=\frac{\int_{0}^{x}I\left(q\right)dq}{\int_{0}^{1}I\left(q\right)dq}$$

One can easily see that the boundary conditions Π(0)=0 and Π(1)=1 are satisfied.

With two other definitions,

(20) $$G\left(x\right)=\frac{2\tau_{0}}{\sigma^{2}\left(x\right)I\left(x\right)}\;\mathrm{and}\;c_{A}=\frac{\int_{0}^{1}I\left(q\right)\left[\int_{0}^{q}G\left(k\right)dk\right]dq}{\int_{0}^{1}I\left(q\right)dq},$$

the mean time to absorption (either fixation or loss) becomes,

(21) $$T_{A}\left(x\right)=\int_{0}^{x}\left[I\left(q\right)\int_{0}^{q}G\left(k\right)dk\right]dq-c_{A}\int_{0}^{x}I\left(q\right)dq.$$

At x=0 both terms in the above equation vanish, at x=1 they cancel each other, so $T_{A}\left(0\right)=T_{A}\left(1\right)=0$.

For the time to fixation we define two other quantities,

(22) $$H\left(x\right)=\Pi \left(x\right)G\left(x\right)\;\mathrm{and}\;c_{F}=\frac{\int_{0}^{1}I\left(q\right)\left[\int_{0}^{q}H\left(k\right)dk\right]dq}{\int_{0}^{1}I\left(q\right)dq},$$

and obtain,

(23) $$T_{F}\left(x\right)=\frac{1}{\Pi (x)}\left\{\int_{0}^{x}\left[I\left(q\right)\int_{0}^{q}H\left(k\right)dk\right]dq-c_{F}\int_{0}^{x}I\left(q\right)dq\right\}.$$

For some specific cases of μ(x) and $\sigma^{2}\left(x\right)$, one may find analytic solutions or large‐*N* asymptotic approximations for Π(x), $T_{A}\left(x\right)$ and $T_{F}\left(x\right)$, see  Danino et al. (2018); Danino and Shnerb (2018); Meyer and Shnerb (2018). However, in general scenarios μ(x) and $\sigma^{2}\left(x\right)$ are complicated and the analytic approach may become tedious. Here our aim is to present a general methodology, not to deal with a specific case, and therefore we implement numerical solutions for (19‐23). These numerical solutions are very simple and may be executed easily in all available programming languages such as Matlab, Mathematica, Maple, Python and so on.

## Results

In this section we demonstrate the capabilities of our approach. We first implement Monte‐Carlo simulations in which the environment alternate periodically between different states, and then consider stochastic (random order) cases.

### PERIODIC ENVIRONMENTAL STATES

**Figure 3:**
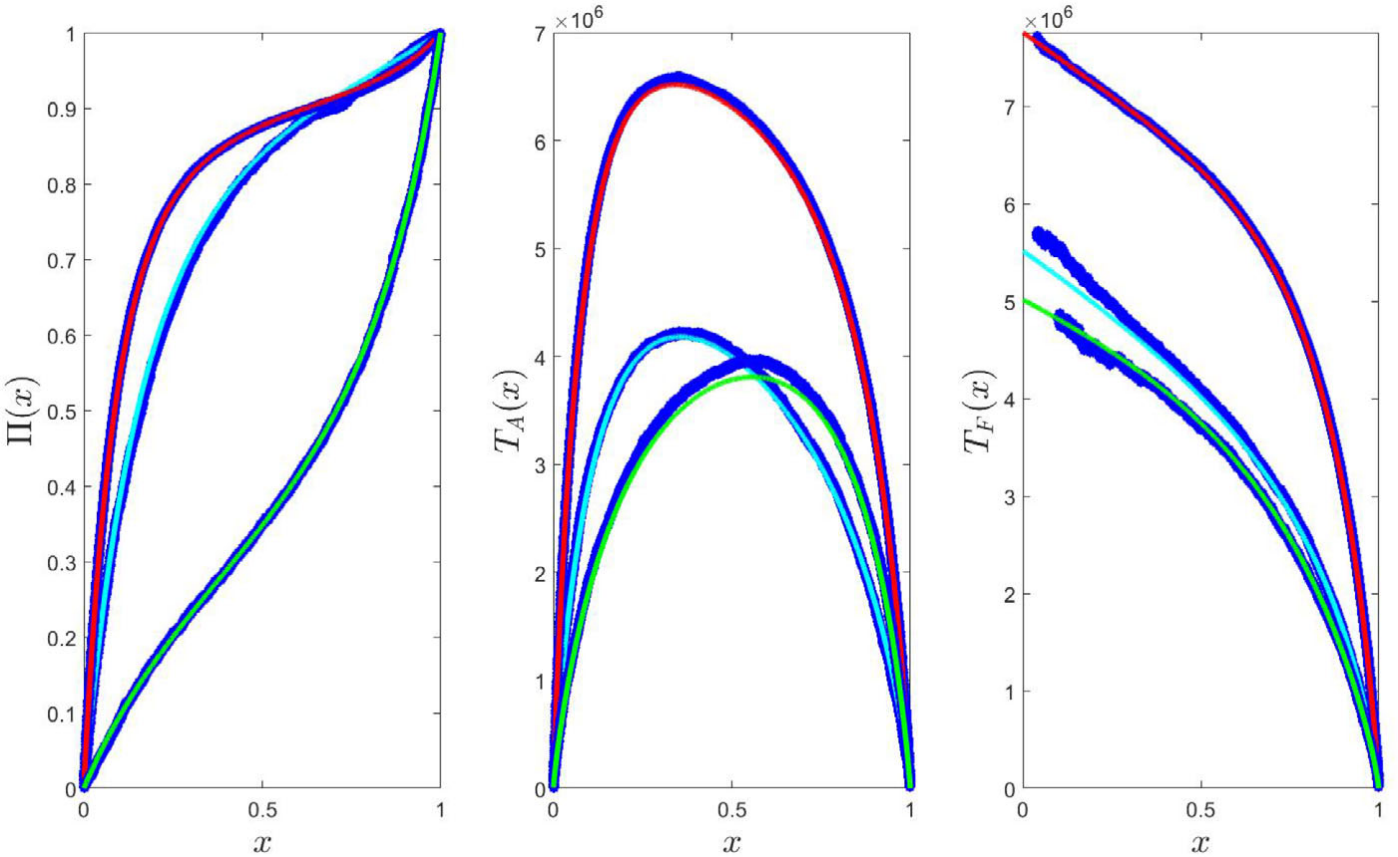
Π(x) (panel a), $T_{A}\left(x\right)$ (panel b) and $T_{F}\left(x\right)$ (panel c) as a function of *x* for various scenarios. Red lines are the relevant numerical solutions of Eqs. (19‐23) for scenario 1 of Table 3. Cyan lines are solutions for scenario 2 and the green lines were obtained for scenario 3. Blue circles are the results of Monte‐carlo simulations in all nine cases (three scenarios, three quantities) and their agreement with the theoretical full lines is evident. Because the chance of fixation of small mutant populations (x→0) is extremely small, the left edge of the $T_{F}$ graphs is quite noisy and we cut some of it. Note that the only difference between scenario 1 and 2 is the type of competition (global or local) in the fixed population low‐abundance case (column ℓ=1 of Table 3), still the results are completely different and our approach manage to predict the outcomes of the numerical experiment.

The simulation results of Figure 3 were obtained from dynamics that are similar to the one presented in Figure 2. Two of the simulations involve four building blocks (constant population, exponential growth, constant population, sharp decline) and one involves only three (constant population, logistic growth, sharp decline) as detailed in Table 3. The order in which these blocks appear is periodic, but the details (for example, the value of γ or the value of *N* when a constant population block started) are not periodic. Figure 3 shows the correspondence between the theoretical prediction [Numerical solutions of Eqs. (19‐23)] and the outcomes of Monte‐Carlo simulations.

**Table 3:** Different scenarios considered in Figure 3

number	block ℓ=1	block ℓ=2	block ℓ=3	block ℓ=4
1	global‐stochastic (i=4) $s_{0}=-0.001$, $r_{0}=1$, γ=0.175, τ=200, δ=100, N=5,000	exponential growth (i=5) $r_{1}=0.01$, $r_{2}=0.00999$ $K_{p}=10,000$, $K_{m}=5,000$	global‐stochastic (i=4) $s_{0}=-0.001$, $r_{0}=1$, γ=0.175, τ=300, δ=100, N=10,000	sharp decline (i=9) $s_{d}=-0.0015$, $t_{sh}=1$ $K_{p}=10,000$, $K_{m}=5,000$
2	local‐stochastic (i=2) $s_{0}=-0.001$, $r_{0}=1$, γ=0.175, τ=200, δ=100, N=5,000	exponential growth (i=5) $r_{1}=0.01$, $r_{2}=0.00999$ $K_{p}=10,000$, $K_{m}=5,000$	global‐stochastic (i=4) $s_{0}=-0.001$, $r_{0}=1$, γ=0.175, τ=300, δ=100, N=10,000	sharp decline (i=9) $s_{d}=-0.00015$, $t_{sh}=1$ $K_{p}=10,000$, $K_{m}=5,000$
3	logistic growth (i=6) $s_{g}=0.015$, λ=0.05 $K_{m}=5,000$, $K_{p}=10,000$	global‐stochastic (i=4) $s_{0}=-0.3$, $r_{0}=1$, γ=0.175, τ=300, δ=100, N=10,000	sharp decline (i=9) $s_{d}=-0.001$, $t_{sh}=1$ $K_{p}=10,000$, $K_{m}=5,000$	–

### RANDOM ORDER OF ENVIRONMENTAL STATES

**Figure 4:**
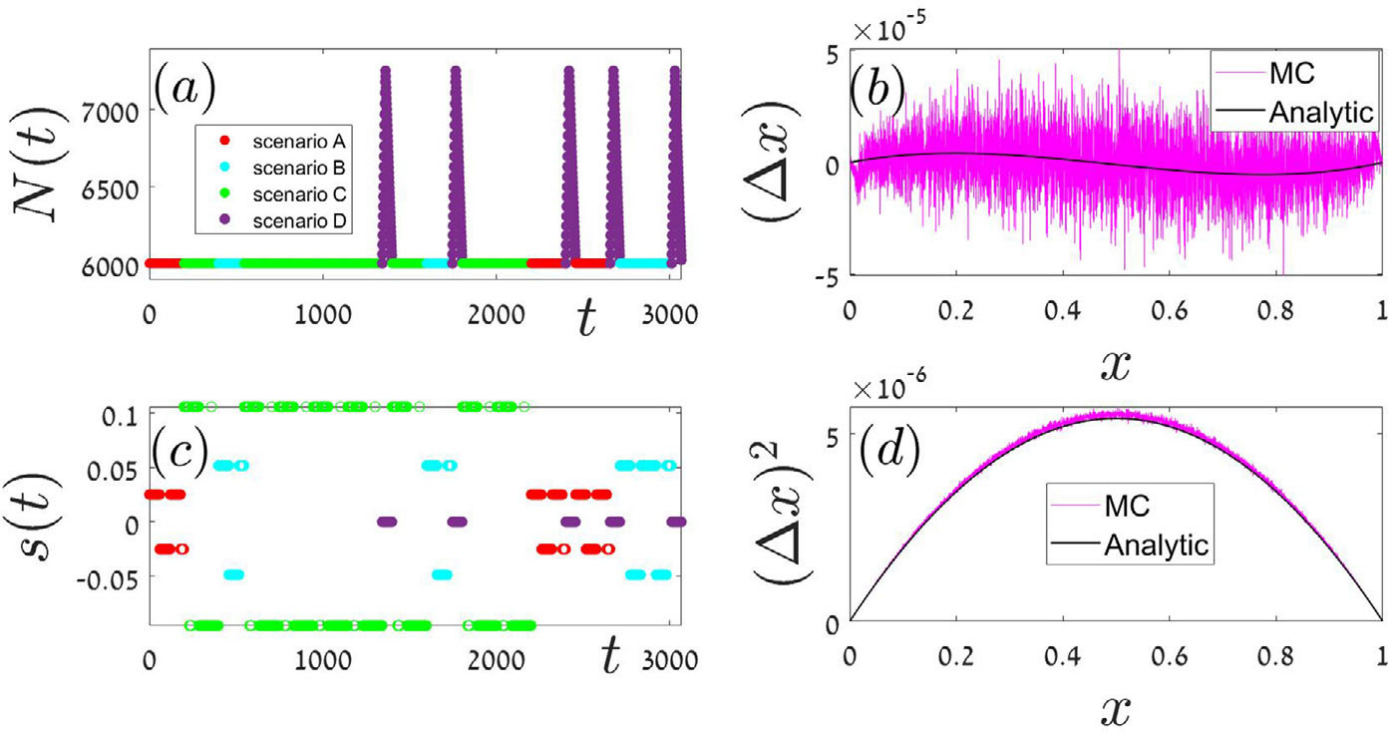
A representative segment made of random combination of the elementary scenarios detailed in Appendix A.4. The four scenarios that were considered in Figures A.1‐A.4 appear at random order. Population size (panel a) and selection parameter (panel b) are colored differently for each scenario, using the color scheme of Appendix A.4. Our theoretical predictions for Δx (panel c) and its second moment (panel d) are based on Eqs. (17) and (14), using the elementary expressions in lines 1,3,5,7 of Table 2.

In our “stochastic” simulations, the building blocks order is random. Here we implement four different scenarios, each with its own building block (or blocks), parameters and duration. In Appendix A.4 each of these scenarios: local periodic competition Fig A.1 global periodic competition Fig A.2, global stochastic Fig A.3, or exponential growth followed by exponential decline Fig A.4 is addressed separately. In each iteration, our Monte‐Carlo algorithm chooses at random a given scenario out of these four options, until fixation or extinction is achieved. As a result, the order on which these blocks appear is random and aperiodic. One specific history, for example, is shown in the two left panels of Fig 4. The mean velocity and variance were calculated according to Eqs. (17) and (14), and the theoretical prediction is compared with simulation in the two right panels of Fig. 4.

**Figure 5:**
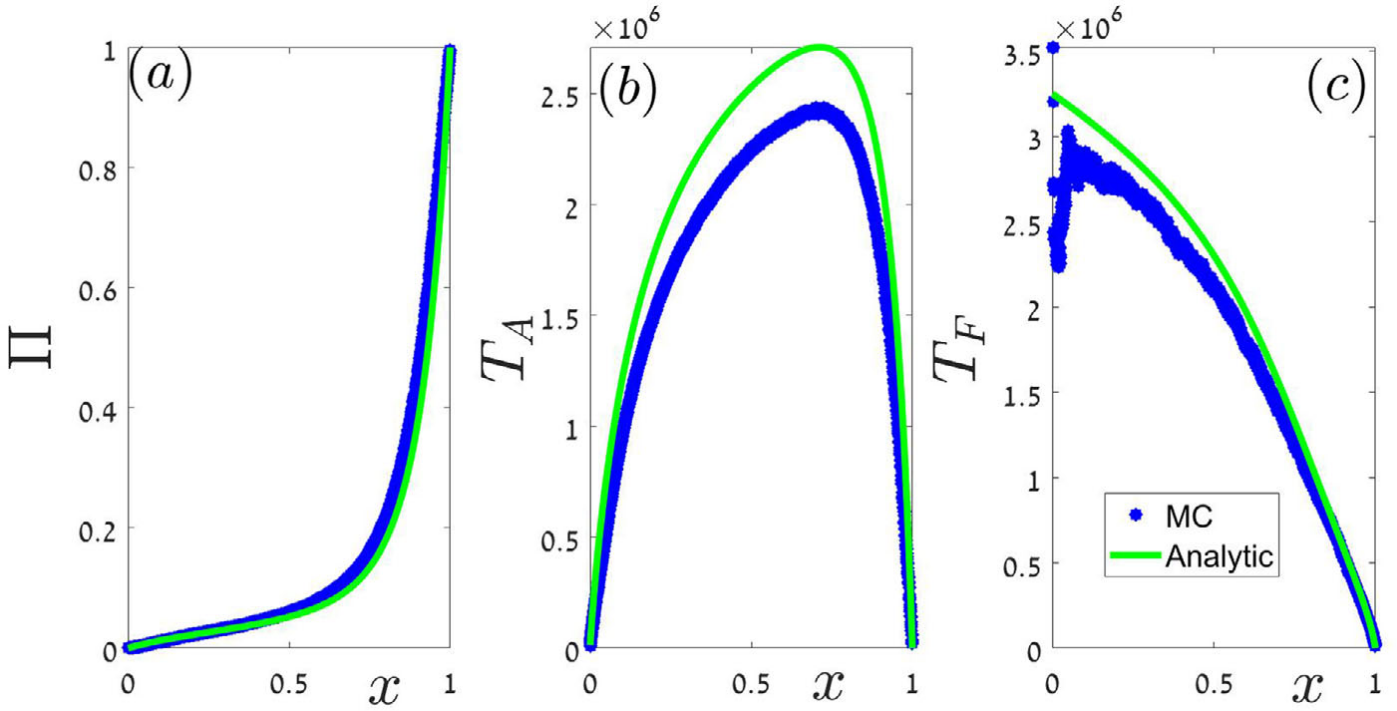
Π(x) (panel a), $T_{A}\left(x\right)$ (panel b) and $T_{F}\left(x\right)$ for a random combination of elementary building blocks. The building blocks are those used in Figure 4, but the simulation is much longer and stochastic, after each step the next building block is picked at random from scenarios A−D of Appendix A.4 with equal probability. The agreement between the outcomes of long Monte‐Carlo simulations (blue) and the theoretical predictions based on the representative segment (green) is quite good.

**Figure A.1:**
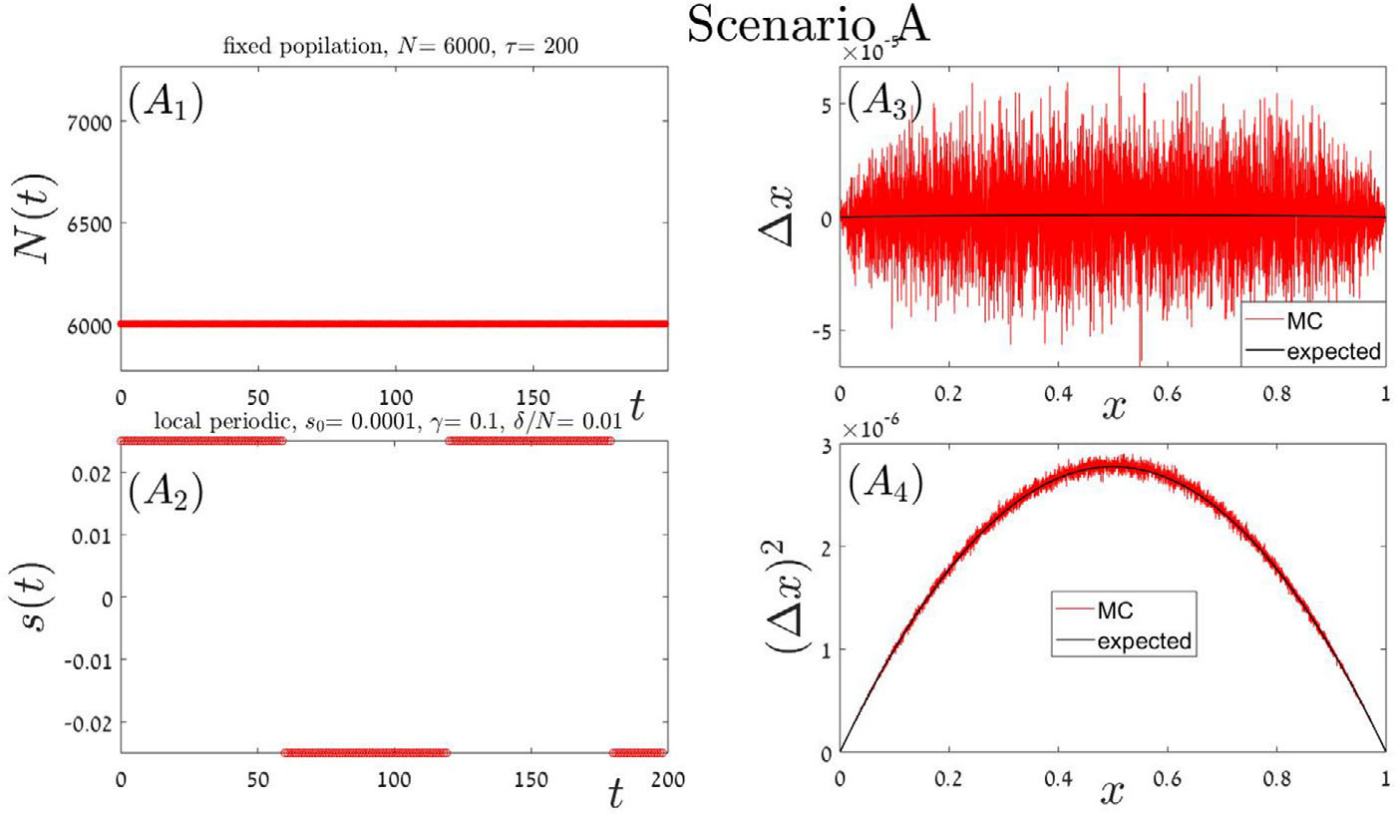
Scenario A: a single block of type i=1. The duration of this block is $\tau =200\tau_{0}$, where τ_0_ corresponds to the mean time required for an elementary birth‐death events (duel), so r=1. The total population size (mutant plus wild type) is stable at N=6000 (panel A1), and the chance of the mutant type to win a duel is given by Eq. (5) where $s=s_{1}-s_{2}$ jumps periodically between two values, as indicated in panel (A2). The mean displacement as a function of *x* (panel A3), as well as the second moment (panel A4), as obtained from numerical simulations of the process (red), are compared with the results presented in the first line of Table 2.

**Figure A.2:**
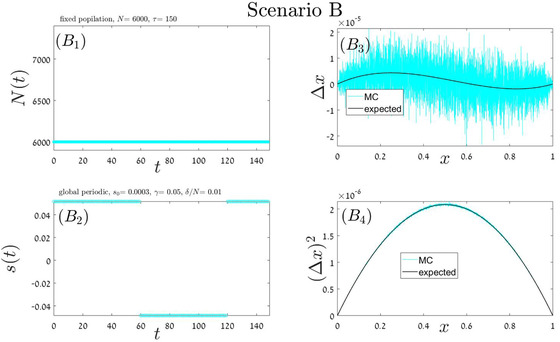
Scenario B: a single block of type i=3. The duration of this block is $\tau =150\tau_{0}$, where τ_0_ corresponds to the mean time required for an elementary birth‐death events of global competition (Moran process), so r=1. The total population size (mutant plus wild type) is stable at N=6000 (panel B1), and the chance of the mutant type to win an empty slot is given by Eq. (8) where $s=s_{1}-s_{2}$ jumps periodically between two values, as indicated in panel (B2). The mean displacement as a function of *x* (panel B3), as well as the second moment (panel B4), as obtained from numerical simulations of the process (cyan), are compared with the results presented in the third line of Table 2.

**Figure A.3:**
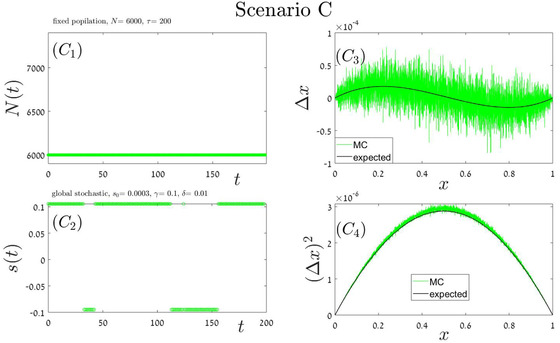
Scenario C: a single block of type i=4. The duration of this block is $\tau =200\tau_{0}$, where τ_0_ corresponds to the mean time required for an elementary birth‐death events of global competition (Moran process), so r=1. The total population size (mutant plus wild type) is stable at N=6000 (panel C1), and the chance of the mutant type to win an empty slot is given by Eq. (8) where $s=s_{1}-s_{2}$ jumps stochastically between two values, as indicated in panel (C2). The mean displacement as a function of *x* (panel C3), as well as the second moment (panel C4), as obtained from numerical simulations of the process (green), are compared with the results presented in the forth line of Table 2.

**Figure A.4:**
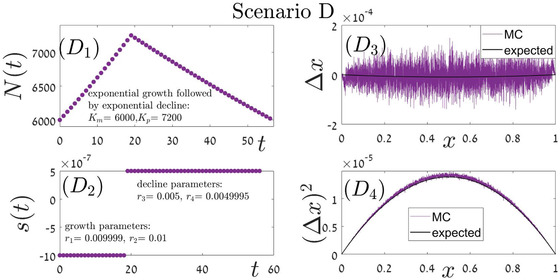
Scenario D is a combination of two building blocks. First, the population grows exponentially, with *r*_1_ for the mutant, *r*_2_ for the wild type, until $K_{p}$ is reached. This corresponds to line i=5 of Table 2. Then, the population decreases exponentially, with *r*_3_ for the mutant and *r*_4_ for the wild type, in a way that corresponds to line i=7 of Table 1. The overall duration of the process depends on the initial *x* value, and panels D1 (total population size) and D2 (fitness differences, expressed by $r_{1}-r_{2}$ in the growth phase and by $r_{3}-r_{4}$ in the decline phase) correspond to the case of x=1/2. Our theoretical predictions for Δx (panel D3) and its second moment (panel D4) are based on Eqs. (17) and (14), using the elementary expressions in lines 5 and 7 of Table 2.

Once μ(x) and $\sigma^{2}\left(x\right)$ were calculated, we substitute these quantities into Eqs. (19‐23)]. Numerical solutions of the corresponding integrals are compared with the outcomes of long runs of our Monte‐Carlo simulations in Figure (5).

## Discussion

For many years, the diffusion approximation serves as the standard tool in the analysis of stochastic processes in general and population genetics in particular (Wakeley, 2005). Despite its limitations, it yields surprisingly accurate results when the relevant rates are slow enough and provides qualitative or semi‐quantitative insights even beyond its range of applicability (Danino et al., 2018; Parsons et al., 2010).

In the classical works of Kimura and co‐workers, the diffusion approximation was applied to processes in fixed environments, but until recently only a few cases of fluctuating environments were considered (Takahata et al., 1975; Takahata & Kimura, 1979). The interest in the effects of environmental variations on population processes grows substantially during the last decade, and many authors dealt with fluctuating selective forces or with variations in population size (Ashcroft et al., 2014; Cvijović et al., 2015; Danino and Shnerb, 2018; Huerta‐Sanchez et al., 2008; Hidalgo et al., 2017; Mustonen and Lässig, 2008; Meyer and Shnerb, 2018; Marrec & Bitbol, 2020; Wienand et al., 2017, 2018). Again the diffusion approximation was implemented, but this has been done in an ad‐hoc manner and the relevant terms $\sigma^{2}\left(x\right)$ and μ(x) were calculated from scratch in each case.

This paper aims at providing a simple mechanistic algorithm that allows one to produce and solve DA equations. It is based on the reduction of any complicated history into a sequence of basic building blocks. This approximation is supposed to work when the fine details of the dynamic are not crucial. If these fine details are crucial, the problem is less interesting, as its solution requires accurate knowledge of the history and cannot provide any general insights. We believe that the arsenal of simple building blocks considered here—local and global competition, logistic and exponential growth and decline, stochastic and periodic fluctuations and sharp decline— is wide enough so one may replace the observed or predicted process by a representative segment made of these building blocks. Once such a segment is defined, the $\mu_{i}\left(x\right)$ and $\sigma_{i}^{2}\left(x\right)$ terms for each block may be read from Table 2 and then combine, using the method presented in Section 4, to yield the relevant diffusion equation, whose numerical (and sometimes even analytic) analysis is quite trivial.

As explained, the main restriction on our approach is the requirement that *x* changes only slightly in the duration of a single representative segment. When this is not the case the diffusion approximation breaks down. Clearly, when the timescale of environmental variations is much larger than the fixation time the fate of the mutant depends mainly on the environmental state when the mutation occurs. This is the “quenched” limit considered by Mustonen and Lässig (2008); Cvijović et al. (2015). In particular, when the dynamics allow for bottlenecks and the total population size *N* drops to a small number, one expects the diffusion approximation to fail. Another scenario in which this problem manifests itself is when the environmental variations are superimposed on trends that are longer than $T_{F}$.

In  Meyer and Shnerb (2020), schematic model of competition in varying environment was analyzed, and the same quantities were considered (Π(x), $T_{A}\left(x\right)$ and $T_{F}\left(x\right)$). Here, we expand this work in two important manners. First, we included Table 2 that provided the relevant information for many building blocks, only a few of them were considered in Meyer and Shnerb (2020). More importantly, the analysis of Meyer and Shnerb (2020) is limited to the first‐order effect in selection parameters (neglects terms like $s_{0}^{2},g,s_{g}s_{0}$ and so on). Here we included second order terms both in the calculations of the building block contribution (for example the term $g=\gamma^{2}\delta /\left(2N\right)$ in line 4 of Table 2) and in the equations used to combine the effect of individual blocks (Eqs. 16 and 17). To the first order in selection parameters $\mu_{m}\left(x\right)$ and $\sigma^{2}\left(x\right)$ are both simple additive sums of the contributions of all building blocks; only when higher orders are considered the non‐commutative characteristics of building blocks (Eq. 16) must be taken into account.

Keeping second order terms in the selection parameters is crucial to the analysis of fluctuating environments. When only linear terms are kept, dynamic is governed by the mean selective force. In that case [as done in Meyer and Shnerb (2020)] the original fixed environment expressions describe the long‐term behavior of the system. Environmental variations may affect the mean values of the effective selection parameters, they cannot yield dynamics that differ qualitatively from the dynamics in fixed environment. Only when higher order terms are taken into account, new qualitative behaviors like the storage effect and relative nonlinearity (Chesson & Warner, 1981; Chesson, 1982a, 2000; Dean & Shnerb, 2020; Ellner et al., 2016; Letten et al., 2018; Usinowicz et al., 2017) manifest themselves. These effects have to do with the covariance between selection parameters and population size, or with nonlinear terms stabilize coexistence and protects polymorphism, but they are essentially nonlinear so to capture their influence one must include *s* ^2^ terms. In principle, the analysis presented here may be extended to include third‐order and higher‐order terms in the selection parameter, but we are not familiar with important effects that appear only at this level. Moreover, if higher‐order terms are really important then selection terms have to be relatively large and in that case the diffusion approximation itself may break down.

The distinction between stochastic and periodic environmental variations is relevant to two distinct points. First, in the constant population dynamics (blocks 1 − 4 of Table 2) we distinguished between periodic and stochastic selection variations. As explained in  Meyer et al. (2022), periodic systems are more stable (admit higher $T_{A}$) than their stochastic counterparts, as they do not allow for rare sequences of bad years (long periods of negative selection). Second, in the assembly of many building blocks into a representative segment one must take periodicity into account. If the dynamic is periodic (i.e., if the order and duration of building blocks is periodic) then the natural candidate for a representative segment is one period. On the other hand when building blocks appear in a stochastic order and/or duration there is no natural recipe like that, and one must identify a reasonable representative segment for the dynamics (see Figure 1). The chosen segment does not have to be periodic. Note that the two aspects of stochasticity and periodicity, the one within block and the one involves the blocks themselves, are completely independent: the representative segment may include periods of constant population with stochastic variations even if the order of blocks is periodic and periodic constant population blocks even if the order of blocks is stochastic.

As pointed out by Wakeley  (Wakeley, 2005), the role of the diffusion approximation in the theory of population genetic is much more than just a technical tool. As the only generally applicable analytic technique, it played an important role in shaping the conceptual framework of the field. Since environmental fluctuations and variations in population size and selective forces are so common in nature and even in experimental systems, a simple and generic procedure that allows one to implement the diffusion approximation in these cases is definitely required. We believe that the scheme presented here provides such a platform, and hope it will contribute to further developments in this important research field.

## AUTHOR CONTRIBUTIONS

N.M.S was the originator of the concept, and supervised the work of B.S and I.M.. B.S derived the analytical approximations, presented in the appendix (A,B) and section (IV ) of the main text. I.M performed the numerical simulations and provided the figures. The writing was done mostly by N.M.S and I.M.

Associate Editor: S. Charlat

Handling Editor: T. Chapman

## Data Availability

All data generated or analyzed during this study are included in this published article (and its supplementary information files).

## Appendix A Calculating the $\mu_{i}\left(x\right)$ and the $\sigma_{i}^{2}\left(x\right)$ for the Different Scenarios

In this appendix we derive the expressions presented in Table 2 of the main text. Before doing that we would like to present two technical remarks.

First, in what follows $B_{n}\left(p\right)$ is a random number drawn from a binomial distribution with *n* trials and success probability *p*.

Second, in some cases we would like to divide a random variable *f* (whose mean is $\bar{f}$) by another random variable *g* (mean $\bar{g}$). In general one may write the expected value of the quotient as:

(A.1)
$$E\left[\frac{f}{g}\right]=E\left[\frac{\bar{f}+\left(f-\bar{f}\right)}{\bar{g}+\left(g-\bar{g}\right)}\right]=E\left[\frac{1}{1+\frac{\left(g-\bar{g}\right)}{\bar{g}}}\frac{\bar{f}+\left(f-\bar{f}\right)}{\bar{g}}\right].$$

If the distribution of *g* around its mean is such that $|g-\bar{g}|<\bar{g}$ (at least with high enough probability), a Taylor series expansion yields,

$$E\left[\frac{f}{g}\right]\approx E\left[\frac{\bar{f}+\left(f-\bar{f}\right)}{\bar{g}}\left(1-\frac{\left(g-\bar{g}\right)}{\bar{g}}+\frac{{\left(g-\bar{g}\right)}^{2}}{{\bar{g}}^{2}}\right)\right]\approx$$

(A.2)
$$\frac{\bar{f}}{\bar{g}}\left(1+\frac{E\left[{\left(g-\bar{g}\right)}^{2}\right]}{{\bar{g}}^{2}}\right)-\frac{1}{{\bar{g}}^{2}}E\left[\left(f-\bar{f}\right)\left(g-\bar{g}\right)\right],$$

such that the quotient of the means is a first approximation for the mean of the quotient. The validity of this approximation may be verified by estimating the variance of *g* and the correlation between *f* and *g*.

Table 2 provides the results for nine scenarios. The first four are constant population cases (periodic and stochastic, global and local competition) and the derivation of the appropriate expressions is presented in Meyer et al. (2022). In the other five cases total population varies and we consider them here. First the easiest problem (sharp decline) followed by the logistic and the exponential growth or decline cases.

### SHARP DECLINE (LINE 9 OF TABLE 2)

Let us consider first the simplest case, a sharp decline (dilution) of the total population from $K_{p}$ to $K_{m}$ individuals. In that case the new population of the mutant type (1) and the wild type (2) are given by the random variables:

(A.3)
$$n_{1}=B_{xK_{p}}\left(s_{d}+\frac{K_{m}}{K_{p}}\right)\;\text{and}:\;n_{2}=B_{\left(1-x\right)K_{p}}\left(\frac{K_{m}}{K_{p}}\right)$$

and the effect of the decline on the mutant type frequency is:

(A.4)
$$x\to \frac{n_{1}}{n_{1}+n_{2}}.$$

Therefore,

(A.5)
$$\Delta x=\frac{\left(1-x\right)n_{1}-xn_{2}}{n_{1}+n_{2}}.$$

Now we implement Eq. A.2 with $f=\left(1-x\right)n_{1}-xn_{2}$ and $g=n_{1}+n_{2}$. Notice that $\bar{g}\approx K_{m}$ is suppose to be a very large number, and the fluctuations $g-\bar{g}$ are proportional to $\sqrt{K_{m}}$ which is much smaller. Accordingly we may neglect the other terms and estimate,

(A.6)
$$\mu_{i=9}\left(x\right)\approx \frac{1}{t_{sh}}\frac{x\left(1-x\right)\left[\left(s_{d}Kp+K_{m}\right)-K_{m}\right]}{K_{m}}=s_{d}x\left(1-x\right)\frac{Kp}{K_{m}}\frac{1}{t_{sh}}$$

where $t_{sh}$ is the duration of the abrupt decline in τ_0_ units. This duration time goes to zero, but (as explained in the main text) the actual parameter in the diffusion equation is $\mu_{i=9}\left(x\right)$ multiplied by $t_{sh}$, so $t_{sh}$ cancels out and plays no role in the analysis. The approximation of large $K_{m}$ would also allow us to ignore the variance of $n_{1}+n_{2}$ when calculating the second moment:

$$\begin{array}{ccc} & & \sigma_{i=9}^{2}\left(x\right)\\ & & \approx \frac{{\left(1-x\right)}^{2}x\left(s_{d}Kp+K_{m}\right)\left(K_{p}-s_{d}Kp-K_{m}\right)+x^{2}\left(1-x\right)K_{m}\left(K_{p}-K_{m}\right)}{t_{sh}K_{p}K_{m}^{2}}= \end{array}$$

and since $s_{d}x{\left(1-x\right)}^{2}\left(2K_{m}-K_{p}\left(1-s_{d}\right)\right)$ is usually smaller than $\mu^{2}\left(x\right)$, we may simplify and write the variance as:

(A.7)
$$\sigma_{i=9}^{2}\left(x\right)\approx \frac{1}{t_{sh}}x\left(1-x\right)\left[\frac{1}{K_{m}}-\frac{1}{K_{p}}\right]$$

### EXPONENTIAL GROWTH AND DECLINE (LINES 5 AND 7 OF TABLE 2)

We consider a birth‐only (death only) process where each timestep dt is measured in units of τ_0_. In case of growth,

(A.8)
$$\Delta n_{1}\equiv B_{xN}\left(r_{1}dt\right)\;,\;\;\Delta n_{2}\equiv B_{(1-x)N}\left(r_{2}dt\right),$$

and an exponential decline yields,

(A.9)
$$\Delta n_{1}\equiv -B_{xN}\left(r_{3}dt\right)\;,\;\;\Delta n_{2}\equiv -B_{(1-x)N}\left(r_{4}dt\right).$$

From now on we consider only growth, the case of exponential decline is completely symmetric.

The change in mutants frequency *x* is,

(A.10)
$$x\to \frac{x+\Delta n_{1}/N}{1+\Delta n_{1}/N+\Delta n_{2}/N}.$$

where *N* is the instantaneous population size. Therefore the displacement in each step is:

(A.11)
$$\Delta x=\frac{x+\Delta n_{1}/N}{1+\Delta n_{1}/N+\Delta n_{2}/N}-x=\frac{\left(1-x\right)\Delta n_{1}/N-x\Delta n_{2}/N}{1+\Delta n_{1}/N+\Delta n_{2}/N}.$$

Taking $f=\left(1-x\right)\Delta n_{1}/N-x\Delta n_{2}/N$ and $g=1+\Delta n_{1}/N+\Delta n_{2}/N$ in Eq. A.2, and assuming that the process is smooth enough, i.e. $r_{i}dt\ll 1$, then $\bar{g}∼1$ and $|g-\bar{g}|$ is much smaller than $\bar{g}$. Therefore we may approximate the mean displacement by:

(A.12)
$$\mu_{i=5}\left(x\right)=\frac{\left(r_{1}-r_{2}\right)x\left(1-x\right)}{1+r_{1}x+r_{2}\left(1-x\right)},$$

as presented in Table 2.

To find the second moment we can expand the expression for the displacement, Eq. A.11, in a Taylor series (up to the second order in both the $\Delta n_{i}/N$):

(A.13)
$$\begin{array}{ccc} {\left(\frac{x+\Delta n_{1}/N}{1+\Delta n_{1}/N+\Delta n_{2}/N}-x\right)}^{2} & \approx & \\ {\left(\left(1-x\right)\Delta n_{1}/N\right)}^{2}+{\left(x\Delta n_{2}/N\right)}^{2} & - & 2x\left(1-x\right)\frac{\Delta n_{1}\Delta n_{2}}{N^{2}} \end{array}$$

The contribution of the mixed terms to the variance vanishes, as they are uncorrelated. Assuming $1-r_{i}dt\approx 1$, the second moment during a single step dt is,

(A.14)
$$\bar{\Delta x^{2}}={\bar{\Delta x}}^{2}+\frac{1}{N(t)}\left[r_{1}x{\left(1-x\right)}^{2}+r_{2}x^{2}\left(1-x\right)\right].$$

We now have to sum (or integrate) over 1/N(t). *N* grows exponentially and its growth rate is $\left\langle r\right\rangle \equiv r_{1}x+r_{2}\left(1-x\right)$. Starting from N(t=0) ($K_{m}$ for growth) and integrating up to N(t) ($K_{p}$ for growth) one finds:

(A.15)
$$\sum \frac{1}{N(t)}\approx \int_{0}^{t}dt^{\prime }\frac{e^{-\left\langle r\right\rangle t^{\prime }}}{N(t=0)}=\frac{1}{\left\langle r\right\rangle }\left(\frac{1}{K_{m}}-\frac{1}{K_{p}}\right)$$

When the diffusion approximation holds $\mu^{2}\left(x\right)$ is usually negligible, otherwise:

(A.16)
$$\sigma_{i=5}^{2}\left(x\right)\approx \mu_{i=5}^{2}\left(x\right)+\frac{K_{p}-K_{m}}{K_{p}K_{m}\left\langle r\right\rangle \tau }\left[r_{1}x{\left(1-x\right)}^{2}+r_{2}x^{2}\left(1-x\right)\right]$$

when τ is the duration which we can estimate from the previous approximation: if $K_{p}=K_{m}\exp\left[\left\langle r\right\rangle \tau \right]$ than $\tau \approx \log\left(K_{p}/K_{m}\right)/\left\langle r\right\rangle$.

### LOGISTIC GROWTH AND DECLINE (LINES 6 AND 8 OF TABLE 2)

In the model of exponential growth discussed in the last section there are two populations that grow exponentially in time, the only interaction between them is imposed by the fact that the growth halts when the total population reaches a given ceiling. Logistic growth takes place differently: first the carrying capacity is incremented, and then both populations compete for the newly open slots. Logistic decline implies that carrying capacity decreases, and then competition between nutant and the wild type increases in strength until the population reaches the new carrying capacity. We assume the rate in which the carrying capacity changes λ, to be the rate determining (slow) process, whereas competition is much faster. Therefore, only λ determines the overall growth (decline) time $t_{g}$ ($t_{d}$).

When carrying capacity grows by ΔN individuals, the share of the mutant type is picked from a binomial distribution $B_{\Delta N}\left(x+s_{g}x\left(1-x\right)\right)$, and the wild type population grows by ΔN−B. Upon decline by ΔN individuals, the mutant type population decreases by $B_{\Delta N}\left(x-s_{d}x\left(1-x\right)\right)$, and the wild type population death toll is ΔN−B. Note that in both cases, a positive value of $s_{g},s_{d}$ represents a beneficial mutant. To make the calculations simple we consider from now on only growth process, the calculations in the decline case are completely symmetric.

Accordingly, after an increase by ΔN,

(A.17)
$$\begin{array}{ccc} x & & \to \frac{xN+B_{\Delta N}\left(x+s_{g}x\left(1-x\right)\right)}{N(t)+\Delta N}\;\text{or:}\;\Delta x\\ & & \approx \frac{B_{\Delta N}\left(x+s_{g}x\left(1-x\right)\right)}{N(t)}, \end{array}$$

so,

(A.18)
$$\bar{\Delta x}=\Delta Ns_{g}x\left(1-x\right)/N\left(t\right).$$

For small steps we implement the differential form,

(A.19)
$$\frac{dx}{x(1-x)}=s_{g}\frac{dN}{N(t)}.$$

Integrating both sides, when the initial value of *x* is *x*_0_ and *N* is integrated from $K_{m}$ to $K_{p}$, one obtains,

(A.20)
$$\begin{array}{ccc} \log & & \left(\frac{x/x_{0}}{\left(1-x\right)/\left(1-x_{0}\right)}\right)=s_{g}\log\frac{K_{p}}{K_{m}}\to \Delta x\\ & & =\frac{x_{0}{\left(\frac{K_{p}}{K_{m}}\right)}_{g}^{s}}{1-x_{0}+x_{0}{\left(\frac{K_{p}}{K_{m}}\right)}_{g}^{s}}-x_{0}. \end{array}$$

The first moment would be:

(A.21)
$$\mu_{i=6}\left(x\right)=\frac{x\left(1-x\right)\left[{\left(\frac{K_{p}}{K_{m}}\right)}_{g}^{s}-1\right]}{1-x+x{\left(\frac{K_{p}}{K_{m}}\right)}_{g}^{s}}\frac{1}{t_{g}}.$$

The time $t_{g}$ is estimated in Eq. (10) of the main text.

To calculate the second moment we notice that at each step:

(A.22)
$$\bar{\Delta x^{2}}={\bar{\Delta x}}^{2}+\Delta N\left[x+s_{g}x\left(1-x\right)\right]\left[1-x-s_{g}x\left(1-x\right)\right]/N^{2}$$

The $1/N^{2}$ factor makes the whole expression rather small, and by integrating over time we obtained,

(A.23)
$$\sigma_{i=6}^{2}\left(x\right)\approx \frac{1}{t_{g}}\left[\mu_{i=6}^{2}\left(x\right)+x\left(1-x\right)\left(\frac{1}{K_{m}}-\frac{1}{K_{p}}\right)\right].$$

### EXAMPLES

In what follows we provide a few examples. In each of the following figures the two left panels show N(t) and s(t) for a given building block (in the last figure ‐ a combination of two building blocks) whereas the two right panels show Δ(x) and ${\left(\Delta x\right)}^{2}$, as obtained from Monte‐Carlo simulations vs. the prediction that are based on Table 2. In subsection 6.2 of the main text these results are used for the analysis of a stochastic environment model.

## Appendix B Addition of Building Blocks

Addition of the building blocks is related to some elementary features of stochastic dynamics. Let us consider a processes with “velocity” f(x) and *x*‐dependent diffusion constant D(x), both these quantities may be time‐dependent. The value of *x* satisfies the difference equation,

(B.1)
$$x_{t+1}=x_{t}+f_{t+1}\left(x_{t}\right)+\xi_{t}D_{t+1}\left(x_{t}\right),$$

where $\xi_{t}$ is a zero‐mean random variable with unit variance. After T steps one gets:

(B.2)
$$\Delta x\left(T\right)=\sum_{t=1}^{T}\left[f_{t}\left(x_{t-1}\right)+\xi_{t}D_{t}\left(x_{t-1}\right)\right].$$

Expanding $f_{t}$ and $D_{t}$ in a Taylor series around $x_{0}=x\left(t=0\right)$ we get:

(B.3)
$$\Delta x\left(T\right)=\sum_{t=1}^{T}\left[f_{t}\left(x_{0}\right)+\xi_{t}D_{t}\left(x_{0}\right)+\left\{f_{t}^{\prime }\left(x_{0}\right)+\xi_{t}D_{t}^{\prime }\left(x_{0}\right)\right\}\Delta x\left(t-1\right)\right]$$

Now we assume that the period T is divided into *m* intervals, indexed by ℓ=1..m. Each interval lasts for $t_{\ell }$ steps, during which the functional form of f(x) and D(x) is fixed. Dropping the zero subscript, one can write

(B.4)
$$\Delta x\left(T\right)=\sum_{\ell =1..m}\sum_{j=1}^{t_{\ell }}\left[f_{\ell }\left(x\right)+\xi_{j}D_{\ell }\left(x\right)+\left\{f_{\ell }^{\prime }\left(x\right)+\xi_{j}D_{\ell }^{\prime }\left(x\right)\right\}\Delta x\left(t-1\right)\right]$$

If *j* is the *j*‐th step in the *q*s interval then the time after that step is $t=\sum_{\ell =1}^{q-1}t_{\ell }+j$.

At this point we would like to take the mean of Δx. Doing that all ξ terms vanish, and one gets

(B.5)
$$\bar{\Delta x(T)}=E\left[\sum_{\ell =1..m}\sum_{j=1}^{t_{\ell }}\left[f_{\ell }\left(x\right)+f_{\ell }^{\prime }\left(x\right)\Delta x\left(t-1\right)\right]\right]$$

We would like to distinguish between the within‐block and the between‐block dynamic of Δx(t−1) . The mean displacement within block (i.e., the mean displacement of *x* between t=0 and $t=t_{\ell }$, where the values of *f* and *D* are fixed) is given by the inner sum in B.5, so,

(B.6)
$$\begin{array}{ccc} E\left[\Delta x(t_{\ell })\right] & = & E\left[f_{\ell }\left(x\right)t_{\ell }+\sum_{t=1}^{t_{\ell }}f_{\ell }^{\prime }\left(x\right)\sum_{j=1}^{i-1}f_{\ell }\left(x\right)\right]\approx \bar{f_{\ell }\left(x\right)}t_{\ell }\\ & & +\frac{t_{\ell }^{2}}{2}\bar{f_{\ell }^{\prime }\left(x\right)f_{\ell }\left(x\right)}. \end{array}$$

Therefore, the first order approximation for the mean displacement, $\Delta x_{fo}$ (here the subscript implies first order), is given by summing over the contribution of each block, neglecting the displacement of the initial frequency due to former blocks. This will be a simple weighted sum over the effect of each block,

(B.7)
$$\bar{\Delta x_{fo}}=E\left[\sum_{\ell =1\text{…}m}t_{\ell }f_{\ell }\left(x\right)+\frac{t_{\ell }^{2}}{2}f_{\ell }^{\prime }\left(x\right)f_{\ell }\left(x\right)\right].$$

To proceed, one has to specify the distribution from which a given $t_{\ell }$ is picked. We have studied two cases:

- Fixed $t_{\ell }$, so
  $$\bar{\frac{t_{\ell }^{2}}{2}}=\frac{{\bar{t_{\ell }}}^{2}}{2}$$
- $t_{\ell }$ is drawn from exponential distribution with mean $\bar{t_{\ell }}$ :
  $$\bar{\frac{t_{\ell }^{2}}{2}}={\bar{t_{\ell }}}^{2}$$

Therefore, we can conclude (here $\tau_{\ell }\equiv \bar{t_{\ell }}$:

- If $t_{\ell }$ is fixed: $\mu_{\ell }\left(x\right)=f_{\ell }\left(x\right)+f_{\ell }^{\prime }\left(x\right)f_{\ell }\left(x\right)\tau_{\ell }/2$
- If $t_{\ell }$ is drawn from an exponential distribution: $\mu_{\ell }\left(x\right)=f_{\ell }\left(x\right)+f_{\ell }^{\prime }\left(x\right)f_{\ell }\left(x\right)\tau_{\ell }$

Now let us return to Eq. (B.7) and try to improve the approximation. Eq. (B.7) assumes that all blocks have the same starting point. In reality, the second block ℓ=2 starts at the end point of the ℓ=1 block. To account for that, we implement again the series expansion: The mean change in *x* after two consecutive blocks is,

(B.8)
$$\Delta x_{1..2}=x_{2}-x_{0}=\mu_{1}\left(x\right)\tau_{1}+\mu_{2}\left(x\right)\tau_{2}+\mu_{2}^{\prime }\left(x\right)\tau_{2}\mu_{1}\left(x\right)\tau_{1}$$

The first two terms were present in the first order expression $\bar{\Delta x_{fo}}$, the last term accounts for the effect of the displacement during the first block on the displacement due to the second block. Therefore,

(B.9)
$$\mu_{1..2}=\frac{\Delta x_{1..2}}{\sum \tau }=\frac{\mu_{1}\left(x\right)\tau_{1}+\mu_{2}\left(x\right)\tau_{2}+\mu_{2}^{\prime }\left(x\right)\tau_{2}\mu_{1}\left(x\right)\tau_{1}}{\tau_{1}+\tau_{2}}$$

and Eq. (16) of the main text is a generalization of this expression.

The derivation of the second moment, $\bar{{\left(\Delta x\right)}^{2}}$, starting from Eq. (B.4), is based on the same arguments. However, in the limits discussed in this paper (when diffusion approximation holds), the variance is a simple weighted addition, as in 14. As we show below, all other terms are negligible.

By definition,

(B.10)
$$\begin{array}{ccc} \Delta x^{2}\left(T\right) & = & \sum_{\ell_{1}=1..m,\ell_{2}=1..m}\sum_{j_{1}=1}^{t_{\ell_{1}}}[f_{\ell_{1}}\left(x\right)+\xi_{j_{1}}D_{\ell_{1}}\left(x\right)+\{f_{\ell_{1}}^{\prime }\left(x\right)\\ & & +\xi_{j_{1}}D_{\ell_{1}}^{\prime }\left(x\right)\}\Delta x\left(t-1\right)]\\ & & \times \sum_{j_{2}=1}^{t_{\ell_{2}}}[f_{\ell_{2}}\left(x\right)+\xi_{j_{2}}D_{\ell_{2}}\left(x\right)+\{f_{\ell_{2}}^{\prime }\left(x\right)\\ & & +\xi_{j_{2}}D_{\ell_{2}}^{\prime }\left(x\right)\}\Delta x\left(t-1\right)] \end{array}$$

Taking the expectation value, terms that are linear in ξ disappear and $\xi_{i}\xi_{j}=\delta_{i,j}$. Therefore,

(B.11)
$$\begin{array}{ccc} \bar{\Delta x^{2}\left(T\right)} & = & E\left[\sum_{\ell =1..m}\sum_{j=1}^{t_{\ell }}\left[D_{\ell }^{2}\left(x\right)+D_{\ell }^{\prime }\left(x\right)D_{\ell }\left(x\right)\Delta x\left(t-1\right)\right]\right]\\ & + & E\left[\sum_{\ell_{1}=1..m,\ell_{2}=1..m}\sum_{j_{1}=1}^{t_{\ell_{1}}}\sum_{j_{2}=1}^{t_{\ell_{2}}}\left[f_{\ell_{1}}\left(x\right)f_{\ell_{2}}\left(x\right)+2f_{\ell_{2}}\left(x\right)f_{\ell_{1}}^{\prime }\left(x\right)\Delta x\left(t-1\right)\right]\right] \end{array}$$

For the cases discussed in this paper, for each *x* $D^{\prime }\Delta x\ll D$ and $f^{\prime }\Delta x\ll f$ (Δx is the small parameter in the expansion that yields the diffusion approximation). Since our models assume no correlation between blocks, mixed terms like $f_{i}f_{j}$ contribute only if *i* and *j* are at the same block. Eq. (B.11) then reduces to,

(B.12)
$$\begin{array}{ccc} E\left[\Delta x^{2}\left(T\right)\right] & = & E\left[\sum_{\ell =1..m}\sum_{j=1}^{t_{\ell }}\left[D_{\ell }^{2}\left(x\right)+t_{\ell }\bar{f_{\ell }^{2}\left(x\right)}\right]\right]\\ & & =\sum_{\ell =1..m}\bar{t_{\ell }}D_{\ell }^{2}\left(x\right)+\bar{t_{\ell }^{2}}\cdot \bar{f_{\ell }^{2}\left(x\right)} \end{array}$$

Where $\bar{t_{\ell }^{2}}$ is either ${\bar{t_{\ell }}}^{2}$ (fixed) or $2{\bar{t_{\ell }}}^{2}$ (stochastic), as discussed above.

Finally, dividing by the total duration of all blocks:

(B.13)
$$\sigma_{1\text{…}m}^{2}\left(x\right)=\frac{\sum_{\ell =1..m}\bar{t_{\ell }}D_{\ell }^{2}\left(x\right)+\bar{t_{\ell }^{2}}\cdot \bar{f_{\ell }^{2}\left(x\right)}}{\sum_{\ell =1..m}\tau_{\ell }},$$

and taking into account that $\sigma^{2}\gg \mu^{2}$, one recovers Eq. (14) of the main text,

(B.14)
$$\sigma_{m}^{2}\left(x\right)=\frac{1}{T_{m}}\sum_{\ell =1..m}\sigma_{\ell }^{2}\left(x\right)\tau_{\ell }.$$

## Appendix C Solutions for the Diffusion Equation

Here we would like to show the integral‐solution for the equation

(C.1)
$$\frac{\sigma^{2}\left(x\right)}{2}F^{\prime \prime }\left(x\right)+\mu \left(x\right)F^{\prime }\left(x\right)=G\left(x\right).$$

when calculating the chance of fixation, F(x)=Π(x) and G(x)=0, for the time to absorption $F\left(x\right)=T_{A}\left(x\right)$ and $G\left(x\right)=-\tau_{0}$, while for the time to fixation we define $Q\left(x\right)=T_{F}\left(x\right)\Pi \left(x\right)$. In this case, F(x)=Q(x) and $G\left(x\right)=-\Pi \left(x\right)\tau_{0}$. We add an integrating factor, such that,

(C.2)
$${\left[F^{\prime }\left(x\right)e^{\int^{x}\frac{2\mu (t)}{\sigma^{2}\left(t\right)}dt}\right]}^{\prime }=\frac{2G(x)}{\sigma^{2}\left(x\right)}e^{\int^{x}\frac{2\mu (t)}{\sigma^{2}\left(t\right)}dt}.$$

Integrating both sides yields,

(C.3)
$$\begin{array}{c} F^{\prime }\left(x\right)e^{\int^{x}\frac{2\mu (t)}{\sigma^{2}\left(t\right)}dt}=\int^{x}\frac{2G(t)}{\sigma^{2}\left(t\right)}e^{\int^{t}\frac{2\mu (q)}{\sigma^{2}\left(q\right)}dq}dt+C_{1}\\ F^{\prime }\left(x\right)=\frac{\int^{x}\frac{2G(t)}{\sigma^{2}\left(t\right)}e^{\int^{t}\frac{2\mu (q)}{\sigma^{2}\left(q\right)}dq}dt+C_{1}}{e^{\int^{x}\frac{2\mu (t)}{\sigma^{2}\left(t\right)}dt}}. \end{array}$$

Finally,

(C.4)
$$F\left(x\right)=\int^{x}\frac{\int^{k}\frac{2G(t)}{\sigma^{2}\left(t\right)}e^{\int^{t}\frac{2\mu (q)}{\sigma^{2}\left(q\right)}dq}dt+C_{1}}{e^{\int^{k}\frac{2\mu (t)}{\sigma^{2}\left(t\right)}dt}}dk+C_{2}$$

All that is left is to find the constants of integration using the boundary conditions ($T_{A}\left(0\right)=T_{A}\left(1\right)=Q\left(0\right)=Q\left(1\right)=\Pi \left(0\right)=0$, and Π(1)=1). This yields the expressions presented in the main text.
